# Gasless, endoscopic trans-axillary thyroid surgery: our series of the first 51 human cases

**DOI:** 10.1186/s12957-021-02484-z

**Published:** 2022-01-07

**Authors:** Rong Cong, Xinying Li, Hui Ouyang, Wenbo Xue, Zeyu Zhang, Fada Xia

**Affiliations:** Department of General Surgery, Xiangya Hospital, Central South University, No. 87 Xiangya Road, Changsha, 410008 China

## Abstract

**Background:**

The safety of gasless endoscopic trans-axillary thyroid surgery is still undetermined.

**Methods:**

Clinical findings and postoperative complications of patients who had undergone trans-axillary thyroid surgery due to thyroid cancer and thyroid nodules were retrospectively studied. The sensory change and paralysis results from this technique and patients’ satisfaction with the cosmesis were also studied.

**Results:**

Fifty-one patients (49 females and 2 males) received operations by gasless, endoscopic trans-axillary approaches with one patient whose operation was converted to open surgery because of internal jugular vein injury. Only two patients developed temporary vocal cord paralysis and no patients developed other severe complications. The alleviation of the discomfort in the anterior neck area and sternocleidomastoid, and the cosmetic effect of gasless endoscopic trans-axillary thyroid surgery were acceptable. No evidence of recurrence was found during the follow-up period.

**Conclusions:**

Gasless, endoscopic trans-axillary thyroid surgery is a feasible procedure with acceptable safety and better cosmetic results in strictly selected patients.

## Introduction

Thyroid cancer is an increasingly prevalent malignancy worldwide [1]. Surgery is the most commonly used technique in treating thyroid diseases. While the conventional collar incision is widely used, endoscopic thyroidectomy is gradually becoming accepted for thyroid nodule patients with cosmetic needs [2–4]. Under the premise of oncological effectiveness, endoscopic thyroidectomy can provide a safe option for these patients [5]. Trans-axillary robotically assisted approaches were first reported by Lobe et al. The addition of robotics can improve surgical dexterity in a difficult-to-reach anatomic region [6]. Duncan et al. reported the endoscopic trans-axillary technique. This procedure ensures patients’ cosmetic needs by making incisions under the axilla, which allows for the removal of nodules, even large nodules [7]. Based on this procedure, Kang et al. further described gasless endoscopic thyroidectomy through the axillary region, which can effectively avoid the influences of carbon dioxide [8]. Several reports with large case cohorts were regarding robotically assisted thyroidectomy [8–10]. However, the da Vinci system is blamed for a prolonged surgery duration and a significantly higher economic cost. Thus, gasless endoscopic thyroidectomy without the assistance of robots might be more suitable. In this study, we reported our initial experiences with 51 patients who underwent gasless endoscopic trans-axillary thyroid surgery performed by a single experienced surgeon. Clinical findings and postoperative complications were analyzed. Furthermore, the sensory change and paralysis results from this technique and patients’ satisfaction with the cosmesis were also studied.

## Materials and methods

### Patients’ enrollment

Patients diagnosed with thyroid cancer and thyroid nodules with diameters > 4 cm who underwent trans-axillary thyroid surgery from July 2020 to March 2021 in Xiangya Hospital were retrospectively enrolled. The inclusion criteria were as follows: (1) patients with thyroid nodules diagnosed as papillary thyroid cancer (PTC) by ultrasound-guided fine needle aspiration (FNA) before surgery—lobectomy (LT) with prophylactic unilateral (ipsilateral) central neck dissection (CND) was performed for these patients; (2) PTC patients with central lymph nodes that were evaluated as negative by two individual professional ultrasound physicians; and (3) patients with benign unilateral nodules with diameters > 4 cm that needed surgical intervention. The exclusion criteria were as follows: (1) patients with benign nodules with diameters > 6 cm, (2) patients who needed a total thyroidectomy, and (3) patients for whom endoscopic surgery was not suitable due to past medical history of cervical and chest wall surgeries or other reasons. Fifty-one patients (42 PTC patients and 9 thyroid adenoma patients) were included in this study. This study was reviewed and approved by the Ethics Committee of Xiangya Hospital, Central South University (No. 202011960).

### Surgical procedures

**Fig. 1:**
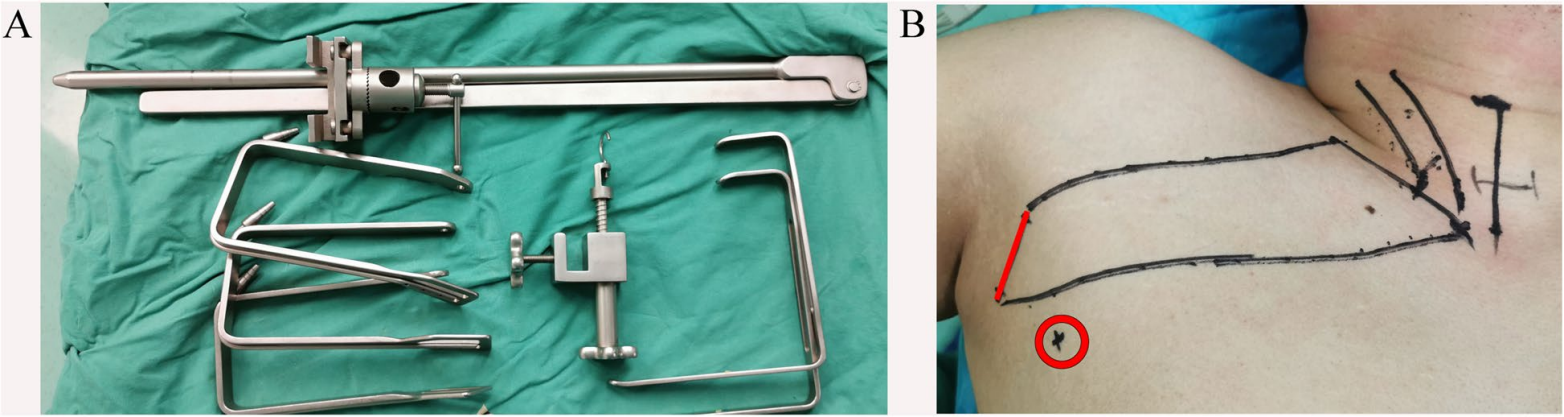
Mechanical retractors and incision selection. **A** The retractors were used to maintain the working space during the operation. **B** A 4-cm length incision was made in the natural wrinkle of axilla and an accessory 5-mm incision was made for another operative instrument

**Fig. 2:**
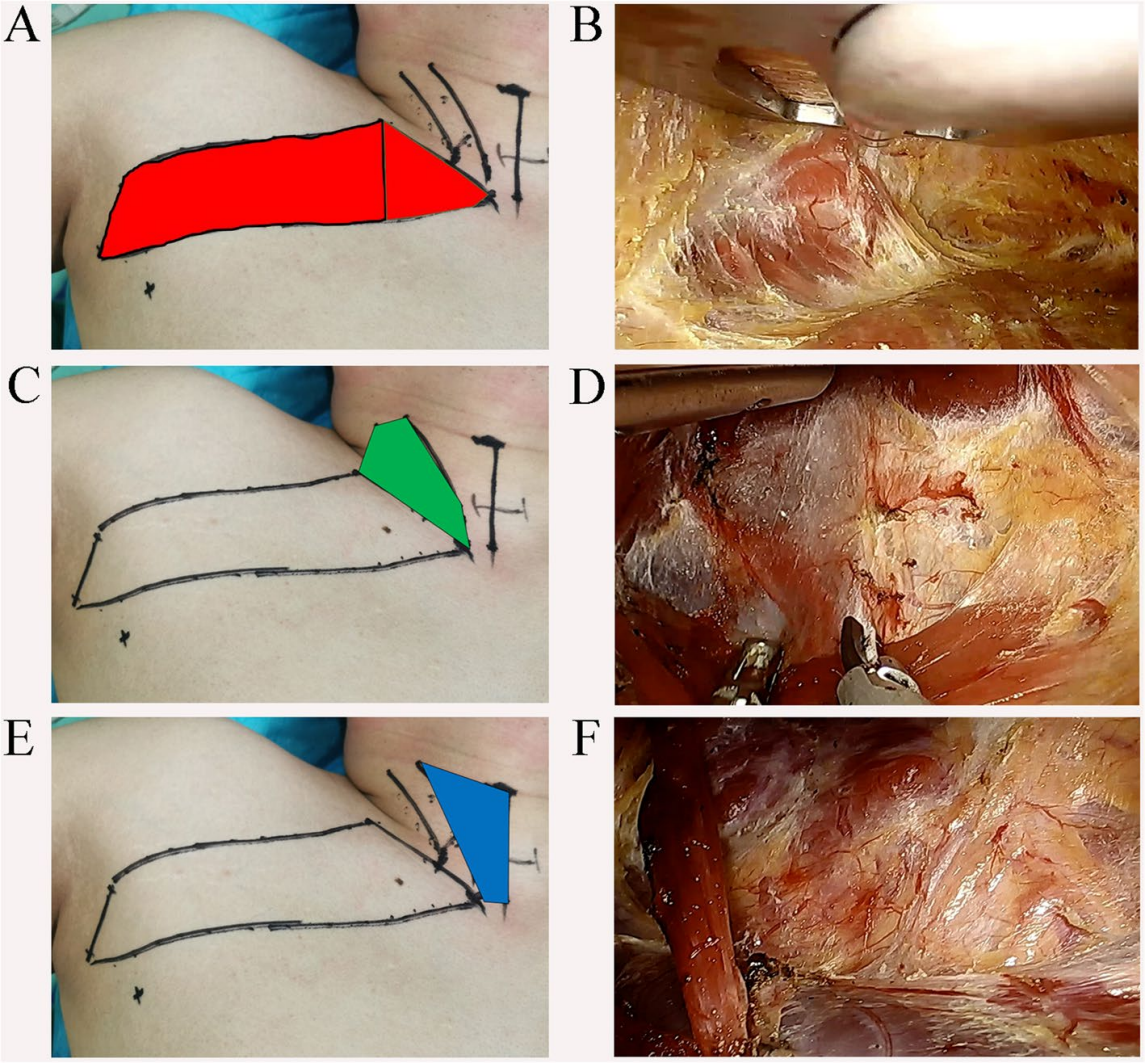
Three standard steps method for working space make. **A**, **B** Step one, dissection of the route to the anterior neck area. The dissected area is marked in red. **C**, **D** Step two, sternocleidomastoid muscle (SCM) was dissected longitudinally and the sternal head of SCM was elevated by the retractor to expose the strap muscles. The dissected area is marked in green. **E**, **F** Step three, the strap muscles were dissected laterally and the retractor was placed beneath the straps muscle to expose the thyroid lobe. The dissected area is marked in blue

**Fig. 3:**
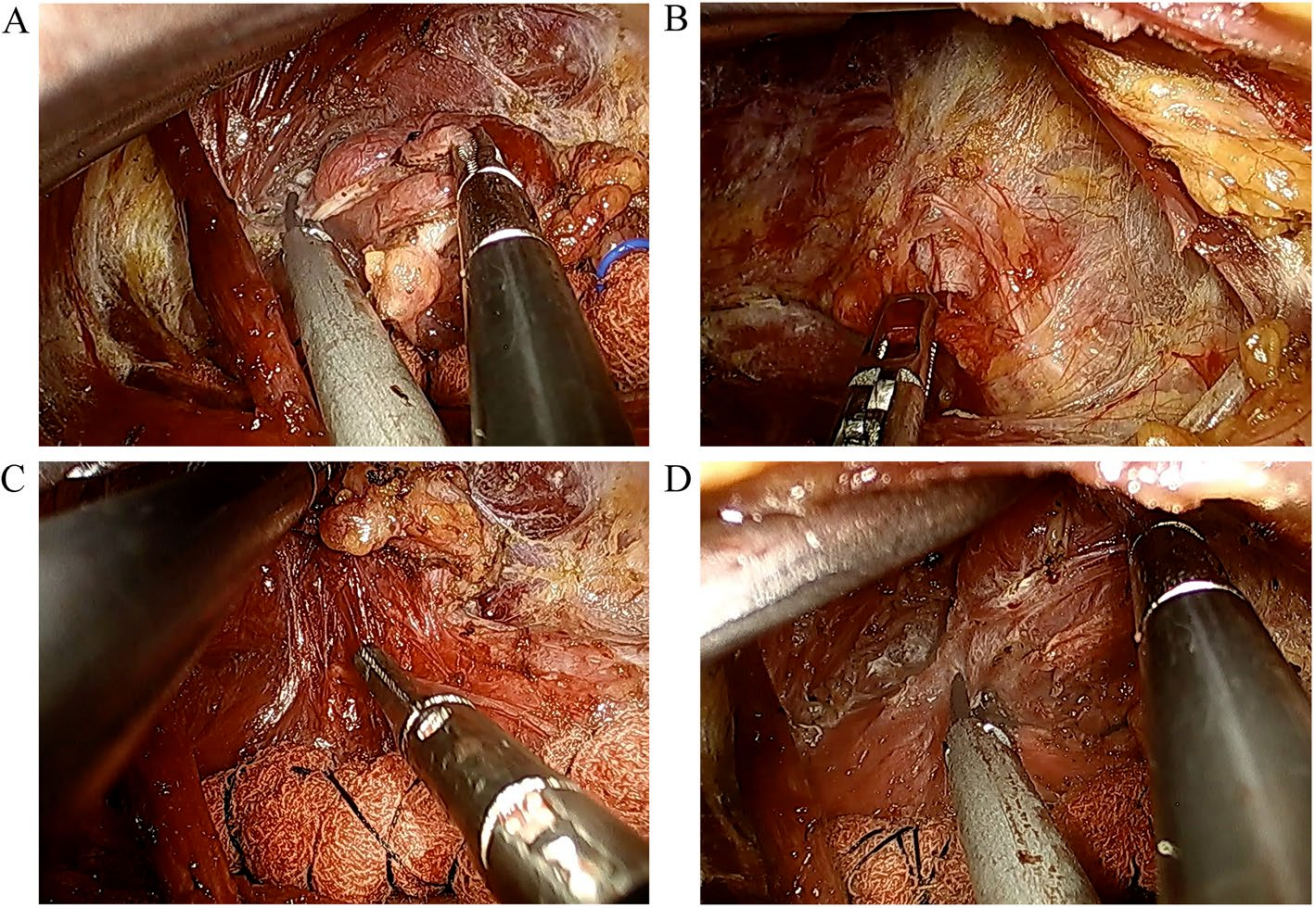
The procedure of thyroidectomy and central neck dissection (CND). **A** The superior thyroid vessels were individually ligated. **B**, **C** The recurrent laryngeal nerve (RLN) was identified and then traced. Prophylactic unilateral central compartment lymph node clearance was conducted. **D** Thyroid isthmus was dissected

The patients were placed in a supine position while the lesion side limb was extended 90 degrees after general anesthesia. A 4-cm length incision was made just behind the anterior axillary fold for the laparoscope (a rigid 30-degree videoscope) and one operating instrument. The retractors (Kangji, Hangzhou, China) were used to maintain the working space during the operation. An accessory 5-mm incision was made on the vertical line of the axillary incision (3–5 cm far away from this incision) for another operating instrument (Fig. 1). The surgical procedure was similar to the methods described by other surgeon groups [11, 12]. As the working space is crucial to the surgery, we have summarized this process in three standard steps. First, we dissected the tunnel to the anterior neck area with exposure of the sternocleidomastoid muscle (SCM). Second, we divided the area between the sternal and clavicular head of the SCM, up to the annular cartilage and down to the clavicle. The sternal head of the SCM was subsequently retracted to expose the strap muscles. Last, the strap muscles were dissected laterally, the omohyoid muscle and internal jugular vein (IJV) were also exposed. Retractor was placed beneath the strap muscles to finish creating the working space (Fig. 2). The procedure for thyroidectomy and CND was similar to that of other endoscopic and open approaches. The middle thyroid vein was then ligated. Prophylactic unilateral lymphadenectomy was also routinely performed among patients with thyroid cancer including the prelaryngeal, pretracheal, and ipsilateral paratracheal areas (Fig. 3).

### Patients’ surveillance

Medical records were reviewed retrospectively. The follow-up times were 3 to 10 months. A questionnaire regarding the sensory changes around the anterior chest, paralysis of the SCM, and satisfaction with cosmesis was used to evaluate the quality of life in enrolled patients. The neck appearance score (also known as the patient satisfaction score, PSSs) was applied to investigate the cosmetic effects. The PSSs were 1 (very satisfied), 2 (satisfied), 3 (unsatisfied), or 4 (very unsatisfied) [13]. Verbal response scores (VRSs) for sensory changes around the anterior chest and paralysis of the SCM (1-week, 1- and 3- month visits) were as follows: 0 = Never, 1 = Sometimes, 2 = Most of the time, 3 = All the time [14].

### Statistical analysis

Continuous variables are presented as the mean ± SD and the mean (range). Statistical analyses were conducted using the Student’s *t* test and one-way ANOVA through the SPSS 23.0 software. A *P* value < 0.05 was considered as statistically significant.

## Results

From July 2020 to March 2021, 51 patients (49 females and 2 males) underwent operations by gasless, endoscopic trans-axillary approaches, with one patient whose operation was converted to open surgery because of internal jugular vein injury. The mean age was 34 years old, ranging from 19 to 45 years. Nine patients had solitary or cystic thyroid adenoma, and thus, lobectomy was performed. Preoperative fine-needle aspiration cytology (FNAC) suggested malignancy in 42 patients, where lobectomy and prophylactic unilateral central neck dissection were performed. The location of the tumor was right, *n*= 30; left, *n*= 21. The mean size of the benign tumors was 39 mm (range 31–49 mm), and the mean size of malignant tumors was 10,0 mm (range 5–26 mm) (Table 1).

**Table 1:** Demographic data and operative details

Characteristics	Value
Age (mean, range)	35 (22–48)
Gender	
Male	2
Female	49
Thyroid disease	
PTC	42
Thyroid adenoma	9
Tumor size (mm, mean, range)	
Benign tumor	39 (31–49)
Malignant tumor	10.0 (5–26)
Location of tumor	
Right lobe	30
Left lobe	21
Extent of surgery	
LT+ CND	42
LT	9
Operative time (mins, mean ± SD)	
Working space make	44 ± 12.8
LT with or without CND	40.9 ± 15.1
Total surgical time	141.6 ± 34.4
Blood loss (mL, mean ± SD)	21.0 ± 26.8
Drainage volume (mL, mean ± SD)	
Day 1	48.0 ± 24.4
Day 2	13.7 ± 5.6
Drainage removal (days)	2
Removed lymph nodes^a^	3.15 ± 3.2
Hospital stay after surgery (days, mean, range)	1.67 ± 0.74 (1–3)

*PTC* papillary thyroid carcinoma, *LT* lobectomy, *CND* central neck dissection

^a^Lymph nodes removed in central neck compartment in PTC cases

**Fig. 4:**
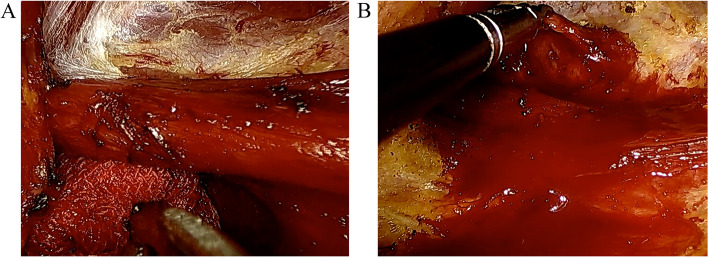
Injury of internal jugular vein (IJV) in two cases. **A** This case has been converted to open surgery. **B** In this case, the hemorrhage of IJV has been controlled by using Harmlock

The total mean operative time was 141.6 ± 34.4 min (range 95–240 min, from anesthetic intubation to leaving the operating room and entering the postanesthetic care unit (PACU)). As the working space is crucial to the surgery and time-consuming, the time of working space makes and thyroidectomy were calculated separately. The total mean operative time of working space make was 44 ± 12.8 min (range 20–120 min). The time of LT with or without CND was 40.9 ± 15.1 min (range 30–90 min). The average blood loss was 21.0 ± 26.8 ml (10–200 ml). The average drainage volume was 48.0 ± 24.4 ml (20–100 ml) on day one and 13.7 ± 5.6 ml (10–30 ml) on day two. The surgical drain was removed 2 days after surgery in all cases. The mean number of lymph nodes removed by unilateral CND was 3.15 ± 3.2 (range 2–12) in PTC patients. The recurrent laryngeal nerves were clearly identified and preserved in all cases. Only two patients developed temporary vocal cord paralysis and no patient developed permanent vocal cord paralysis. None of the patients developed hypoparathyroidism, esophageal injury, or surgical site infection after the surgery. The median hospital stay was 1.67 ± 0.74 (range 1–3) days. The internal jugular vein was injured in two of the patients during the making of working space, one patient’s operation was converted to open surgery. In the other case, the hemorrhage of IJV has been controlled by using Harmlock clips (Fig. 4).

Compared with conventional open surgery, gasless endoscopic trans-axillary thyroid surgery requires the dissection of the anterior neck area and splitting the SCM, which has been described in the endoscopic lateral neck dissection [15]; however, the stiffness and paraesthesia of the SCM have not been evaluated. Sensory changes around the anterior chest and paralysis of the sternocleidomastoid muscle were evaluated by VRSs. VRSs were relatively high at the 1-week and 1-month visits and were significantly decreased at the 3rd-month visit. This suggests that discomfort in the anterior neck area and SCM is obvious shortly after surgery and is relieved over time in most cases. We divided the patients into two groups in surgical order to compare the VRSs regarding paralysis of the SCM. The VRSs in a short time (1 week) were significantly higher in the first 25 cases than in the last 25 cases (the patient whose operation was converted to open surgery was excluded). As the sternal head of the SCM was elevated by the retractor during the procedure, the decreased VRSs may be partly due to the shorter surgical time and improved surgical techniques. The neck appearance score was low in all cases (either satisfied or very satisfied). The cosmetic effect and the self-healing of the discomfort in the anterior neck area and SCM suggest that the application of gasless endoscopic trans-axillary thyroidectomy is feasible. No evidence of recurrence was found during the follow-up period.

## Discussion

Remote access is focused by many surgeons in the decade to be a feasible technique in thyroidectomy avoiding the neck scar caused by conventional surgery, which includes endoscopic or robotic approaches [16, 17]. In this study, 51 patients accepted gasless, endoscopic trans-axillary thyroid surgery with only one patient whose operation was converted to open surgery. Due to the working space needed for the flap formation and division of the SCM during an axillary approach, this technique is more time-consuming. In the first 20 cases, the longest time of working space makes was more than 2 h. However, it is likely that the operative time, including working space makes and thyroidectomy with or without CND, will gradually decrease as we gain more experience.

Lee et al. revealed that trans-axillary thyroidectomy was associated with a better cosmetic index, however, it was also associated with a longer operation time, aggravated voice changes, paresthesia, and changes in swallowing function [18]. Piccoli et al. also reported a study concerning the gasless trans-axillary thyroidectomy with the assistance of robots. And their results showed this procedure was feasible with an acceptable safety profile where the need of conversion to conventional thyroidectomy was found in only 2 (0.45%) cases. No internal jugular vein injury was reported [19]. It seems no consensus has been reached on the clinical application of this technique considering the discrepant complications rate. In our study, only two cases had temporary vocal cord paralysis. The complications, including hypoparathyroidism, tracheal injury, esophageal injury, or surgical site infection did not occur. Although the numbers of identified parathyroid glands have not been calculated, we considered that the recognition and protection of the parathyroid gland would get improvement with the growing experience. Our results revealed the complications rate was relatively low and acceptable (Table 2).

**Table 2:** Postoperative complications and cosmetic outcomes

Characteristics	Value	
Recurrent laryngeal never injury		
Temporary	2	
Permanent	0	
Hypoparathyroidism	0	
Hematoma/seroma	1	
Tracheal injury	0	
Esophageal injury	0	
Surgical site infection	0	
Swallow discomfort	0	
Sensory change around the anterior chest *		
One week	1.33 ± 0.48	
One month	0.51 ± 0.58	
Three months	0.25 ± 0.44	
Paralysis of the sternocleidomastoid muscle (all patients) *		
One week	2.35 ± 0.63	
One month	1.78 ± 0.42	
Three months	0.73 ± 0.45	
Paralysis of the sternocleidomastoid muscle	First 25 cases	Others
One week **	2.72 ± 0.46	2.00 ± 0.57
One month	1.80 ± 0.41	1.77 ± 0.43
Three months	0.88 ± 0.33	0.58 ± 0.50
Cosmetic outcomes		
One week	1.31 ± 0.47	
One month	1.16 ± 0.37	
Three months	1.08 ± 0.39	
Recurrence	0	

*PTC* papillary thyroid carcinoma, *LT* lobectomy, *CND* central neck dissection

*P < 0.001 by one-way ANOVA

**p < 0.001 by Student’s *t* test

It has been pointed out by Kang et al. that the dissected flap width in trans-axillary approach is significantly wider than conventional thyroidectomy, causing greater trauma, and potentially more severe pain [20]. Moreover, the patient’s arm is raised during the entire operation time which might cause pain in the axilla [20, 21]. Our data indicated that sensory changes around the anterior chest are relieved over time in most cases. The stiffness and paraesthesia of the SCM resulting from the division of the SCM are also relieved over time. Meanwhile, the postoperative stiffness and paraesthesia of the SCM can also be ameliorated due to the shorter surgical time, and improved surgical techniques. During the limited follow-up periods, no recurrence has been found. The patients were either satisfied or very satisfied with the cosmetic effects. Despite the additional trauma resulting from the flap formation, the application of the trans-axillary approach is of some value in suitable circumstances. It is noteworthy that there is another obvious disadvantage of this technique. The trans-axillary approach (TA) can hardly locate the contralateral thyroid lobe, causing the limitation of TA in bilateral thyroidectomy. Although researchers made some efforts to achieve it by sectioning the sternohyoid muscle, the long operation duration, pain in the neck, and swallowing discomfort should also be taken into consideration. Bilateral axillary approach for total thyroidectomy ensures better identification of recurrent laryngeal nerve (RLN) and PTG on both sides. However, it also leads to greater trauma [22]. Compared to two-dimensional endoscopic thyroidectomy, three-dimensional endoscopic thyroidectomy is an efficient, safe, and reliable method with better depth perception and stereoscopic vision, and an equally satisfactory outcome [23]. How helpful the three-dimensional endoscopy or robotic endoscopy to the completion of bilateral (total) thyroidectomy via trans-axillary approach is worth further exploration. In conclusion, these results revealed that gasless, endoscopic trans-axillary thyroidectomy is a feasible surgery type with an acceptable safety profile and cosmetic outcomes in strictly selected patients. A more in-depth study of the learning curve of the procedure via the TA approach is urgently needed. Meanwhile, prospective clinical studies are needed to investigate and validate surgical and oncological outcomes.

## Conclusions

Gasless, endoscopic trans-axillary thyroid surgery is a feasible procedure with acceptable safety and better cosmetic results in strictly selected patients.

## Data Availability

All data generated or analyzed during this study are included in this published article.
